# TNFRSF11B Suppresses Memory CD4+ T Cell Infiltration in the Colon Cancer Microenvironment: A Multiomics Integrative Analysis

**DOI:** 10.3389/fimmu.2021.742358

**Published:** 2021-12-06

**Authors:** Jun-rong Zhang, Ping Hou, Xiao-jie Wang, Zong-qi Weng, Xin-chang Shang-guan, Hui Wang, Fang You, Bing-qiang Lin, Zheng-yuan Huang, Xian-qiang Chen

**Affiliations:** ^1^ Department of General Surgery (Emergency Surgery), Fujian Medical University Union Hospital, Fuzhou, China; ^2^ Immunotherapy Institute, Fujian Medical University, Fuzhou, China; ^3^ Department of General Surgery (Colorectal Surgery), Fujian Medical University Union Hospital, Fuzhou, China; ^4^ Department of Emergency Medicine, Fujian Medical University Union Hospital, Fuzhou, China

**Keywords:** TNFRSF11B, immune-related genes (IRGs), immunotherapy, memory CD4+ T cell activation, multiomics

## Abstract

**Background:**

Colorectal cancer is a lethal cancer worldwide. Due to the low tumor mutation burden and low proportion of tumor-infiltrating lymphocytes in the microenvironment of most patients, innovative immunotherapeutic approaches need to be identified.

**Methods:**

Using the TCGA-COAD dataset (n = 514), we identified TNFRSF11B as a prognostic factor of colon cancer. An immunohistochemistry (IHC) dataset (n = 86), 290 single colorectal cancer cells (GSE81861), and 31 paired colon cancer transcriptional datasets were further applied to validate the function of TNFRSF11B, which was confirmed *via* fluorescence-activated cell sorting (FACS) analysis.

**Results:**

A risk score system consisting of eight immune-related genes (IRGs) (FGFR2, ZC3HAV1L, TNFRSF11B, CD79A, IGHV3-11, IGHV3-21, IGKV2D-30, and IGKV6D-21) was constructed to predict the prognosis of colon cancer patients. Only TNFRSF11B was closely correlated with late-stage lymph node metastasis and worse survival outcomes (*p* = 0.010, *p* = 0.014, and *p* = 0.0061). In our IHC dataset, 72.09% (62/86) of the colon cancer patients had TNFRSF11B overexpression with significantly shorter overall survival times (p = 0.072). High TNFRSF11B expression typically had a later TNM stage (*p* = 0.067), a higher frequency of lymph node (*p* = 0.029) and lymphovascular (*p* = 0.007) invasion, and a higher incidence of pneumonia (*p* = 0.056) than their counterparts. The expression of six genes (KRT18, ARPC5L, ACTG1, ARPC2, EZR, and YWHAZ) related to pathogenic *E. coli* infection was simultaneously increased with TNFRSF11B overexpression *via* gene set enrichment analysis (GSEA). These genes are involved in the regulation of the actin cytoskeleton, shigellosis, bacterial invasion of epithelial cells, and Salmonella infection. Finally, only activated memory CD4^+^ T cells (*p* = 0.017) were significantly decreased in the high TNFRSF11B expression group *via* CIBERSORT comparison, which was confirmed by TIMER2.0 analysis of the TCGA-COAD dataset. We also performed FACS analysis to show that TNFRSF11B decreased the infiltration of central memory CD4^+^ T cells and effector memory CD4^+^ T cells in the colorectal cancer microenvironment (all *p <*0.001).

**Conclusion:**

TNFRSF11B acts as a prognostic factor for colon cancer patients and could affect the colon cancer immune response. TNFRSF11B was closely related to lymph node invasion and pathogenic *E. coli*. infection, which may negatively affect memory-activated CD4+ T cell infiltration in colon cancer.

## Introduction

In recent years, colorectal cancer has been the third leading cause of cancer-related death in the United States, with an incidence similar to that in China. Distant metastasis and high rates of recurrence are the two major concerns of clinicians (1). In addition to chemotherapy and radiotherapy, immunotherapy has become the third option available for the suppression of colon tumorigenesis. Immune checkpoint inhibitors, particularly programmed death 1 inhibitors, have been widely applied for the immunotherapy of solid tumors, including non-small-cell lung carcinoma, malignant melanoma and advanced renal cell carcinoma, to induce immune normalization (2). However, only approximately 15% of colorectal cancers, namely, those with microsatellite instability-high (MSI-H) status and mismatch repair deficiency (dMMR), referred to as ‘dMMR-MSI-H’, benefit from treatment with nivolumab due to the high proportion of tumor-infiltrating lymphocytes in these tumors (3). The remaining 85% of colorectal cancers have microsatellite instability-low (MSI-L) status, microsatellite stable (MSS) status, and proficient mismatch repair (pMMR), referred to as ‘pMMR-MSI-L’, and efficient immunotherapeutic strategies for these tumors are still lacking. Due to the low tumor mutation burden and low proportion of tumor-infiltrating lymphocytes in the microenvironment of pMMR-MSI-L tumors, researchers have recently proposed innovative therapeutic approaches, such as bispecific antibodies, T cell checkpoint inhibitors and T cell differentiation molecules (4). Tumor necrosis factor receptor superfamily (TNFRSF) members consisting of TNFR2, glucocorticoid-induced tumor necrosis factor (GITR), TNFRSF4 (OX40), and TNFRSF9 (4-1BB) have been found to enhance T cell responses in the innate and adaptive immune systems as they act as costimulatory signals (5).

In this study, after analysis of immune-related genes in the Cancer Genome Atlas Colon Adenocarcinoma (TCGA-COAD) dataset, we defined TNFRSF11B as an independent risk factor for the prognosis of colon cancers, which is closely related to tumor stage and lymph node metastasis. TNFRSF11B, as a decoy receptor for TNFSF11, had a higher affinity for TNFSF11 than TNFRSF11A and competitively hindered the activation of the TNFSF11–TNFRSF11A pathway (6). TNFRSF11A is widely expressed on the surface of colonic follicle-associated epithelium (FAE) and responds to the stimulatory signal from TNFSF11 on the surface of intrastromal cells to promote the formation of M cells that respond to the presentation of luminal antigens. Conversely, TNFRSF11B distributed on the surface of M cells would in turn restrict FAE differentiation toward the M cell phenotype and activate T cells and DCs in combination with TNFSF11. In conclusion, as an immunosuppressant, TNFRSF11B remodels the immune barrier of the colon mucosal immune barrier to alleviate local inflammation (7). Furthermore, the accumulation of soluble TNFRSF11B in the peripheral blood of colon cancer patients could result in a poorer survival rate, especially in patients with TNM stage III disease (8). Knocking out TNFSF11 and TNFRSF11A in mice impeded the formation of secondary lymph nodes, including lymph nodes and Peyer’s patches (9), and TNFRSF11B acted as a suppressive effector in the recruitment of T cells and activation of DCs, which was supported by the results of a melanoma study that showed that in metastatic lymph nodes, melanoma cells produced large amounts of TNFRSF11B, which mediated the impairment of the interaction between T cells and DCs to dampen immune system activation (10). In our cohort, we also demonstrated that TNFRSF11B enhanced colon cancer cell invasion into the lymphovascular system.

Here, we analyzed four independent datasets at the transcriptional and protein levels to determine the role of TNFRSF11B in the tumorigenesis of colon cancer. Moreover, we assessed the differential role and related pathways of TNFRSF11B in the progression of colon cancer *via* gene set enrichment analysis (GSEA) and pseudotime analysis. Finally, we shed light on the suppressive role of TNFRSF11B in the tumor infiltration of activated memory CD4^+^ T cells in colon cancer.

## Methods

### Data Sources

In this study, four independent cohort samples were included to demonstrate the role of TNRFSF11B in the progression of colon cancer. The transcriptome and clinical characteristics of 514 cases in a colon adenocarcinoma (COAD) dataset were downloaded from the TCGA database (11). Single-cell sequencing data and corresponding single-cell functional status from GSE81861 (12), which included the RNA expression profiles of 290 single CRC cells, were downloaded from CancerSEA (13). The transcriptional levels of TNFRSF11B in different normal tissues were acquired from the Harmonizome database (https://maayanlab.cloud/Harmonizome/) (14). The mRNA expression profiles of thirty-one pairs of colon cancer and normal colon epithelium as counterparts were obtained from samples collected during emergency surgery at the Fujian Medical University Union Hospital from June 2018 to January 2019. The presence of TNFRSF11B in paraffin-embedded colorectal cancer tissues sampled during general surgery was assessed at the Fujian Medical University Union Hospital from January 2013 to December 2017. The study protocol was approved by the Institutional Review Board of the Fujian Medical University Union Hospital, and all patients provided written informed consent. All experiments complied with standard biosecurity and institutional safety procedures. The workflow of this study has been presented in **Supplementary Figure 2**.

### Differential Gene Analysis and Annotation

To determine the immune-related genes (IRGs) and transcription factors (TFs) involved in the progression of colon cancer, analysis of differentially expressed genes between cancer and paired noncarcinoma samples was performed *via* the R software edgeR package (15). A false discovery rate (FDR) <0.01 and a log_2_|fold change| >2 as the criteria values were used to select differential genes. Differentially expressed IRGs and TFs, downloaded from the Immunology Database and Analysis Portal (ImmPort) database (16), were then extracted from all differentially expressed genes. To explore the interactions between IRGs and TFs, a PPI network was constructed based on the STRING database (https://cytoscape.org/). Hub gene analysis of the PPI network was further performed using Cytoscape software version 3.8.2. Functional enrichment analyses were performed *via* the GO and KEGG pathways.

### RNA Sequencing and Gene Set Enrichment Analysis (GSEA)

Total RNA was extracted from 62 colon cancer samples using TRIzol (Invitrogen, Paisley, UK), and mRNA libraries were prepared and sequenced at BGI (HiSequation 2000) (17). Reads were aligned to the human reference genome (GRCh38/hg38) by SOAP2.32. The number of transcripts per million was used to detect gene expression levels. The values of transcripts per million were transformed by log2 and normalized by the Limma R package to remove the batch effect (18). To further elucidate the function of TNFRSF11B in colon cancer, GSEA was performed on a transcriptional dataset in our center for the top 25% and bottom 25% of the expressed genes. C2.cp.kegg.v5.2.symbols.gmt from the KEGG pathway database was selected as the reference gene set. Using a Venn diagram, we determined the common altered signaling pathways between cancer paired noncarcinoma analysis and TNFRSF11B expression analysis strategies (19).

### IHC Staining

Eighty-six patients diagnosed with colon cancer who underwent radical colectomy from January 2013 to January 2017 during emergency surgery at the Fujian Medical University Union Hospital were included in this study. Differential analysis of the protein expression levels of TNFRSF11B in these colon cancer tissues revealed the correlation between TNFRSF11B and colon cancer tumorigenesis. Anti-osteoprotegerin (TNFRSF11B) monoclonal antibody (ab9986, Abcam, UK) was used at a working concentration of 1:200. We built IHC systematic scores, integrating the intensity and percentage of positive cells, to evaluate the expression level of TNFRSF11B. The staining intensity was separated as follows: 0, no staining; 1, light yellow staining; 2, yellow-brown staining; and 3, deep brown staining. The percentage of positive cells was scored as follows: 0, 0–5%; 1, 6–25%; 2, 26–50%; 3, 51–75%; and 4, >75%. The final score was calculated as follows: positive cell score × staining intensity score. Finally, patients were sorted into strong- and weak-TNFRSF11B expression groups, and differential analysis was performed between TNFRSF11B expression and clinicopathological features. To further detect the expression levels of TNFRSF11B in normal lymph nodes, TNFRSF11B expression in normal lymph nodes was reviewed by using the immunohistochemical (IHC) staining data provided in the Human Protein Atlas (http://www.proteinatlas.org/) (20).

### Isolation of Human Samples and Flow Cytometry

Peripheral blood mononuclear cells (PBMCs) were isolated from healthy volunteers using lymphocyte separation medium (Lonza) according to the manufacturer’s instructions. After centrifugation and purification, lymphocytes were obtained. Cells with 1 µg/ml mouse anti-human CD28 PerCP-Cyanine5.5 mAb (eBioscience #45-0088-42) were washed with PBS and 1640 culture medium twice and then incubated in a 96-well plate precoated with 0.2 µg/ml mouse anti-human CD3 mAb Alexa Fluor^®^ 488 (eBioscience #53-0037-41) for one day. Soluble TNFRSF11B was added to the culture medium of the test group, while mIgG was added to the control group. Three days later, the cells were analyzed by flow cytometry. The percentages of subgroups including naïve memory CD4^+^ T cells (CCR7^+^CD45RA^+^; CCR7 mAb (3D12), PE-Cyanine7, eBioscience #25-1979-42; mouse anti-human CD45RA BV421, eBioscience #25-1979-42), central memory CD4+ T cells (CCR7^−^CD45RA^−^) and effector memory CD4+ T cells (CCR7^+^CD45RA^−^) were further assessed by flow cytometry.

### Immune Cell Infiltration Analysis

Our transcriptome data were evaluated by comparing CIBERSORT (21) with the LM22 gene matrix as a reference to identify 22 immune cell subtypes. Microarray probes were replaced with HUGO gene symbols. Genes with multiple probes collapsed to the gene with the highest mean expression. Expression profiles were normalized using fRMA. Using TIMER2.0, we further detected the abundance of immune cells in the microenvironment of TCGA-COAD patient samples and the correlation with TNFRSF11B expression (22).

### Statistics

All graphs and statistical visualization were performed by R software (4.0.1) and Hiplot (https://hiplot.com.cn). Qualitative variables were compared by the χ^2^ test or Fisher’s exact test, and quantitative variables were compared *via* t-tests. Through Kaplan–Meier analysis, the long-term survival outcome was calculated, and the independent risk factors were identified by the Cox proportional hazards regression model.

## Results

### Immune-Related Gene Selection and Annotation

**Figure 1:**
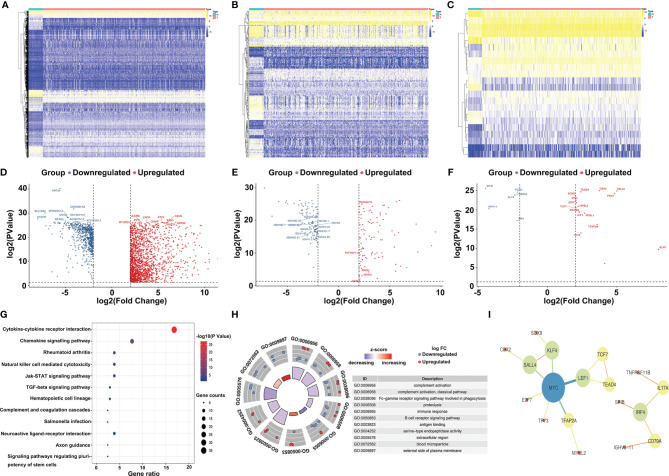
Selection and annotation of differentially expressed immune-related genes. Heatmap **(A)** and volcano plot **(D)** show the significantly (|log2FC| >2 and P value < 0.01) altered genes between tumor and normal tissues, and the top 10 most significant genes in the upregulated group (red dots) and downregulated group (blue dots) are displayed. Similarly, the heatmap **(B)** and volcano plot **(E)** show the significantly different immune-related genes (IRGs), and the heatmap **(C)** and volcano plot **(F)** present the significantly different transcription factors (TFs). Kyoto Encyclopedia of Genes and Genomes pathways (KEGG) **(G)** and gene ontology (GO) **(H)** of differentially expressed IRGs demonstrate the most relevant pathways and gene functions. Protein–protein interactions of hub genes **(I)** between IRGs and TFs were constructed.

A total of 208 immune-related genes (IRGs) (**Figures 1B, E**), including 111 with downregulated expression and 97 with upregulated expression, were identified from the TCGA-COAD database *via* comparison between tumor and normal tissue (n = 472 and 42, respectively), following the analysis of 2,471 differentially expressed genes (**Figures 1A, D**) (|log_2_FC| >2 and P value <0.01). We also identified six downregulated and 16 upregulated differentially expressed genes by the analysis of 318 classical transcription factors (TFs) (**Figures 1C, F**). Functional enrichment analysis demonstrated that these differentially activated IRGs were mostly involved in the cytokine–cytokine receptor interaction KEGG pathway (**Figure 1G**). Furthermore, we revealed twenty-one IRGs closely related to the survival outcome *via* univariate Cox analysis (**Table 1**). These survival-related IRGs were enriched in complement activation, the Fc-gamma receptor signaling pathway involved in phagocytosis, the immune response, the B cell receptor signaling pathway, antigen binding, serine-type endopeptidase activity, the extracellular region and the external side of the plasma membrane based on gene ontology (GO) analysis (**Figure 1H**). We also constructed an interaction network of the survival-related IRGs and TFs (**Figure 1I**). MYC, LEF1, KLF4, SALL4, and IRF4 were the five important TFs involved in the regulation of the immune response in colon cancer. TNFRSF11B may correlate with IL17A in the regulation of the colon cancer immune response.

**Table 1:** Univariate and multivariate analyses of differentially expressed IRGs.

Gene_ID	Univariate		Multivariate	
	HR (95%CI)	*p*	HR (95%CI)	*p*
CD79A	1.016 (1.004–1.027)	0.007	0.974 (0.953–0.995)	0.017
FGFR2	1.086 (1.036–1.139)	0.001	1.073 (1.020–1.130)	0.007
IGHG1	1.000 (1.000–1.001)	0.000		
IGHG3	1.001 (1.000–1.001)	0.001		
IGHG4	1.001 (1.000–1.001)	0.002		
IGHV1-2	1.003 (1.002–1.004)	0.000		
IGHV3-11	1.004 (1.002–1.006)	0.000	1.004 (1.001–1.007)	0.012
IGHV3-21	1.005 (1.003–1.007)	0.000	1.003 (1.000–1.007)	0.041
IGHV4-59	1.003 (1.001–1.004)	0.000		
IGHV6-1	1.027 (1.011–1.044)	0.000		
IGKV2-28	1.028 (1.009–1.048)	0.004		
IGKV2D-28	1.078 (1.030–1.128)	0.001		
IGKV2D-30	1.163 (1.096–1.233)	0.000	1.099 (1.019–1.186)	0.015
IGKV3-20	1.001 (1.000–1.001)	0.002		
IGKV3D-7	1.266 (1.067–1.503)	0.007		
IGKV6-21	1.018 (1.007–1.030)	0.002		
IGKV6D-21	1.043 (1.019–1.068)	0.000	1.035 (1.005–1.065)	0.022
IGLV1-50	1.066 (1.017–1.117)	0.008		
IL17A	1.453 (1.102–1.915)	0.008		
TNFRSF11B	1.010 (1.003–1.017)	0.007	1.008 (1.000–1.016)	0.038
ZC3HAV1L	1.065 (1.020–1.112)	0.004	1.085 (1.037–1.134)	0.000

### Clinical Analysis of Survival-Related IRGs

**Figure 2:**
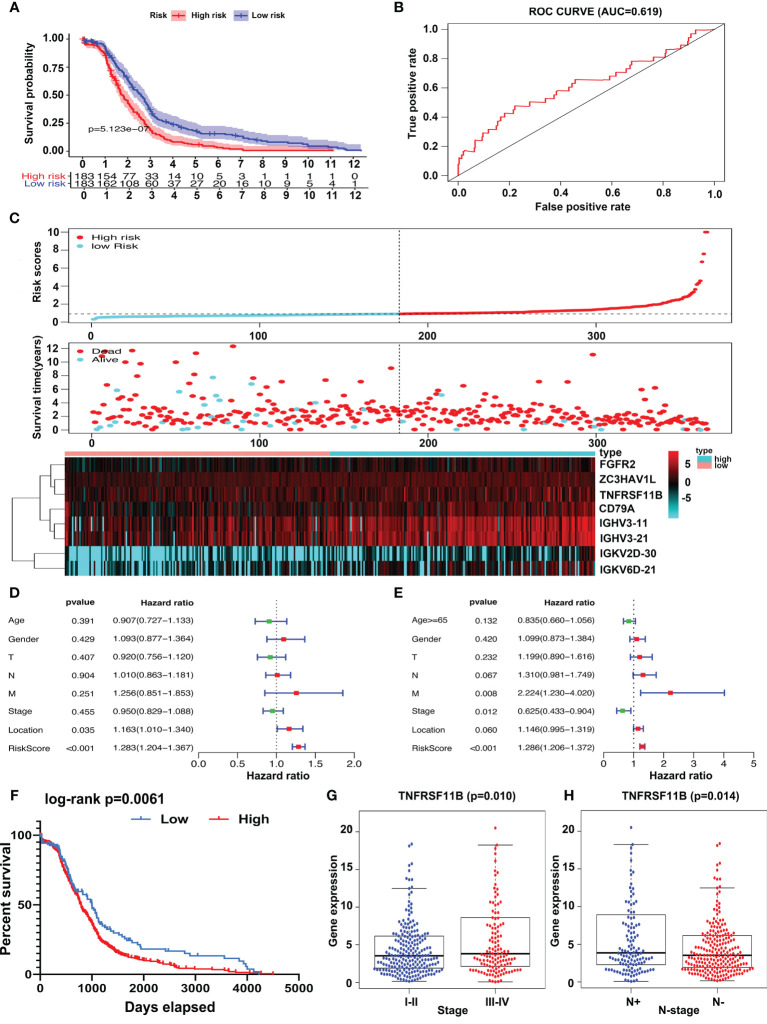
Risk score system construction and clinical characteristic analysis of survival-related IRGs. Risk scoring systems containing FGFR2, ZC3HAV1L, TNFRSF11B, CD79A, IGHV3-11, IGHV3-21, IGKV2D-30, and IGKV6D-21 have been calculated and show that high-risk patients have worse survival outcomes than low-risk patients **(A)**. The area under the curve (AUC) plot **(B)** and prognostic index model **(C)** of the risk scoring systems were determined. Forest plot of univariate **(D)** and multivariate **(E)** analyses of survival-related risk factors. Patients in the TNFRSF11B high‐expression group suffered shorter overall survival intervals **(F)** and a higher incidence of late-stage **(G)** and lymph node metastasis **(H)**.

A risk scoring system consisting of eight IRGs (FGFR2, ZC3HAV1L, TNFRSF11B, CD79A, IGHV3-11, IGHV3-21, IGKV2D-30, and IGKV6D-21) was constructed to evaluate the prognosis of colon cancer patients based on multivariate Cox analysis. Patients were separated into two groups, high risk and low risk, according to the risk scoring system. We demonstrated that the high-risk group had worse overall survival outcomes (p = 5.123∗e^−7^, **Figure 2A**) than the low-risk group, and the area under the curve (AUC) of the receiver operating characteristic (ROC) curve was 0.619 (**Figure 2B**), which supported the potential utility of the risk scoring system. As the risk scores increased, the survival time decreased (**Figure 2C**). We integrated the risk scores with patients’ clinical characteristics, and the risk scoring system was identified as an independent factor *via* univariate and multivariate Cox analyses (**Figures 2D, E**). We analyzed the relationship between the survival-related IRGs and the characteristics of colon cancer patients and found that only TNFRSF11B was strongly associated with TNM stage and lymph node status. Overexpression of TNFRSF11B obviously correlated with late-stage lymph node metastasis and worse survival outcomes (*p* = 0.010, *p* = 0.014, and *p* = 0.0061, respectively) (**Figures 2F–H**).

### Validation of TNFRSF11B in Our Cohort Study

**Figure 3:**
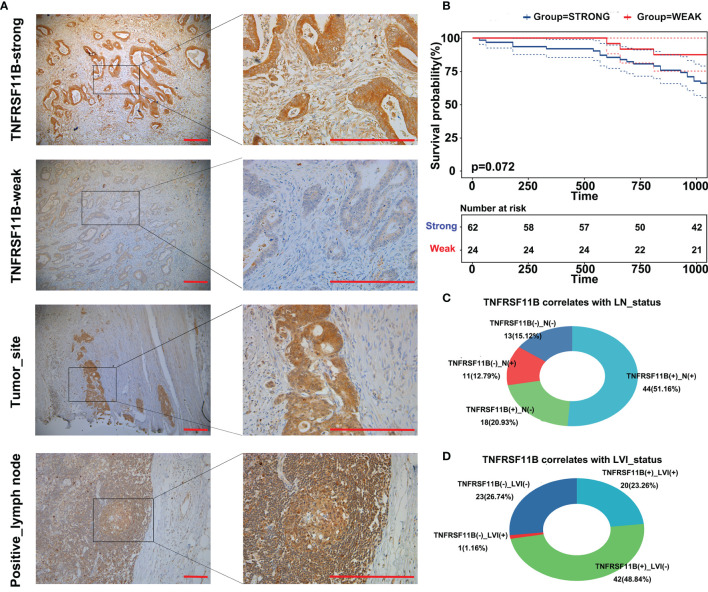
Immunohistochemistry (IHC) staining of TNFRSF11B in our cohort. IHC staining of TNFRSF11B **(A)** strongly and weakly expressed samples, tumor site and metastatic lymph nodes. Patients in the TNFRSF11B-strong expression group suffered shorter overall survival times **(B)**. Correlation between TNFRSF11B expression and the status of lymph node metastasis (LN) **(C)** and lymphovascular invasion (LVI) **(D)**.

To validate the role of TNFRSF11B in the progression of colon cancer, we used immunohistochemistry (IHC) to assess the expression of TNFRSF11B and its correlation with pathological features. In our cohort, 72.09% (62/86) of the colon cancer patients who received radical surgery had TNFRSF11B overexpression, which was associated with significantly shorter overall survival times (p = 0.072, **Figures 3A, B**). Patients with high TNFRSF11B expression typically had a late TNM stage (*p* = 0.067), a high frequency of lymph node (*p* = 0.029) and lymphovascular invasion (*p* = 0.007), and a high incidence of pneumonia (*p* = 0.056) (**Table 2** and **Figures 3C, D**). Conversely, the transcriptional expression of TNFRSF11B in normal lymph nodes remained relatively low levels, *via* data mining in the Harmonizome database (**Supplementary Figure 1A**). Moreover, it’s hard to detect the protein expression of TNFRSF11B in germinal center cells and non-germinal center cells from the Human Protein Atlas database (**Supplementary Figures 1B–D**).

**Table 2:** Comparison of clinicopathological characteristics between the TNFRSF11B-Strong and TNFRSF11B-Weak groups.

Characteristic	Strong-group (n = 62)	Weak-group (n = 24)	*P* value
Age, (y)	60.98 ± 14.75	63.83 ± 14.02	0.418
Female/Male, (%**)**	24 (38.70)/38 (61.30)	10 (41.70)/14 (58.30)	0.801
Size, (cm)	6.12 ± 2.38	5.83 ± 1.83	0.596
Location, (%)			**0.031**
Right-side colon	4 (6.50)	6 (25.00)	
Transverse colon	20 (32.30)	5 (20.80)	
Left-side colon	34 (54.80)	9 (37.50)	
Rectum	4 (6.50)	4 (16.70)	
pTNM stage, (%)			**0.067**
I	3 (4.80)	1 (4.20)	
II	15 (24.20)	12 (50.00)	
III	44 (71.00)	11 (45.80)	
T stage, (%)			0.650
T1	1 (1.60)	0 (0.00)	
T2	2 (3.20)	1 (4.20)	
T3	36 (58.10)	17 (70.80)	
T4	23 (37.10)	6 (25.00)	
N stage, (%)			**0.029**
N0	18 (29.00)	13 (54.20)	
N+	44 (71.00)	11 (45.80)	
Total_LNs	22.60 ± 10.84	18.29 ± 6.50	**0.028**
Positive_LNs	2.98 ± 3.66	1.21 ± 1.92	**0.004**
Histological features, (%)			0.308
Well differentiated	1 (1.60)	1 (4.20)	
Moderately differentiated	47 (75.80)	17 (70.80)	
Poorly differentiated	14 (22.60)	6 (25.00)	
LVI (+)/(-), (%)	20 (32.30)/42 (67.70)	1 (4.20)/23 (95.80)	**0.007**
WBC, (10^^^9)	8.14 ± 3.46	9.19 ± 6.09	0.431
NLR, (ratio)	6.21 ± 5.87	5.89 ± 4.20	0.804
dNLR, (ratio)	1.65 ± 0.38	1.66 ± 0.37	0.858
PLR, (ratio)	249.95 ± 157.07	220.35 ± 84.99	0.267
LMR, (ratio)	3.03 ± 2.70	2.38 ± 1.16	0.254
SII, (ratio)	1,687.88 ± 1,834.95	1,701.05 ± 2,108.69	0.977
CEA, (ng/mL)	11.52 ± 21.25	16.71 ± 32.41	0.393
Pneumonia, (+)/(-), (%)	19 (22.10)/45 (77.90)	2 (8.30)/22 (91.70)	**0.056**

Continued from the above **Table 1**. dNLR, Derived Neutrophil-to-Lymphocyte Ratio; PLR, Platelet-to-Lymphocyte Ratio; SII, Systemic Immune Inflammation Index; LMR, Lymphocyte-to-Monocyte Ratio; LVI, LymphoVascular Invasion. All P-values < 0.05 were considered statistically significant.

All the significantly different variables were presented with bold font.

### TNFRSF11B Correlated With Colon Cancer Differentiation

**Figure 4:**
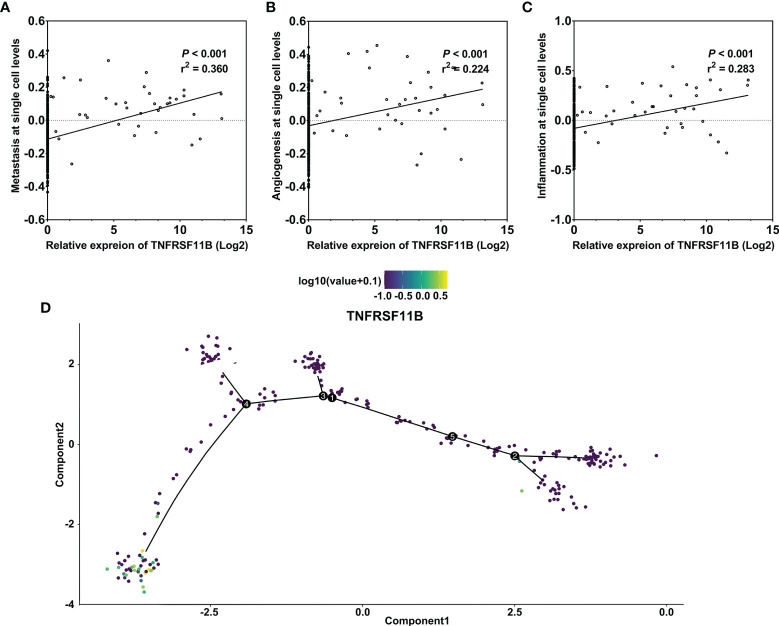
Pseudotime analysis of the function of TNFRSF11B in single colon cancer cells. Correlation between TNFRSF11B expression and the status of metastasis **(A)**, angiogenesis **(B)**, and inflammation **(C)** in single colorectal cancer cells. TNFRSF11B was enriched in the terminal differential stage of colon cancer cells **(D)**.

TNFRSF11B, as a self-regulator of M-cell differentiation in colon mucosa, is also involved in the differentiation of osteoclasts in the bone marrow and secondary lymphoid tissues (7, 9, 23). We analyzed the potential role of TNFRSF11B in the differentiation of colon cancer *via* a GSE81861 array. Increased expression of TNFRSF11B significantly enhanced three cancer-related functional states, namely, metastasis, angiogenesis and inflammation, in colon cancer cells (cor = 0.360, cor = 0.224, and cor = 0283, respectively, all p values <0.001) (**Figures 4A–C**). TNFRSF11B expression was also enriched in the terminal differentiation of colon cancer cells through pseudotime analysis (**Figure 4**). Thus, TNFRSF11B may drive the progression of early-stage colon cells to cells with full tumorigenicity.

### TNFRSF11B Correlated With Pathogenic *E. coli* Infection

**Figure 5:**
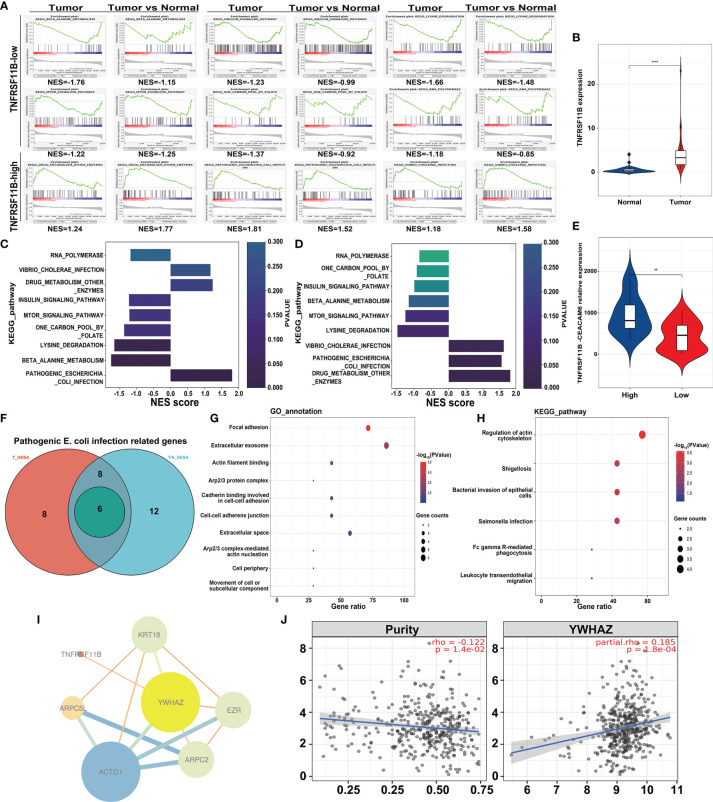
Transcriptome-wide RNA Sequencing to Identify Potential Targets of TNFRSF11B-related pathways in colon cancer samples. Gene set enrichment analysis (GSEA) **(A)** was performed to analyze the common enrichment signaling pathways in different groups (TNFRSF11B-high versus TNFRSF11B-low tumors, tumor versus normal tissues). Gradient histogram showing the common upregulation and downregulation pathways in TNFRSF11B-high tumors **(C)** and a high ratio of TNFRSF11B in the comparison between tumor and normal tissues **(D)**. The expression levels of TNFRSF11B **(B)** and CEACAM6 **(E)** are displayed in a violin plot. A Venn diagram **(F)** shows the core-enriched genes involved in the progression of pathogenic *Escherichia coli* infection, and GO annotation **(G)** and KEGG pathways **(H)** are illuminated. Hub-gene analysis **(I)** based on Cytoscape software shows the correlation between TNFRSF11B and other genes related to pathogenic *Escherichia coli* infection. The correlation between TNFRSF11B and YWHAZ was further confirmed by the TCGA-COAD project **(J)**. **p < 0.01; ****p < 0.0001.

To determine which genes and pathways are responsible for TNFRSF11B expression in colon cancer, we performed transcriptome-wide RNA-sequencing analysis of colon cancer tissues and paired colon epithelium. Two independent gene set enrichment analyses (GSEAs), namely, tumor-only and comparisons between tumors and paired colon epithelium, were applied to identify the involved signaling pathways and genes. Six pathways, including RNA polymerase, one carbon pool by folate, insulin signaling pathway, beta alanine metabolism, mTOR signaling pathway and lysine degradation, were inhibited by the upregulation of TNFRSF11B expression. Pathogenic *Escherichia coli* (*E. coli*) infection, drug metabolism, other enzymes and *Vibrio cholerae* infection were the other three pathways activated by the increased expression of TNFRSF11B. Consistently, only the pathogenic *E. coli* infection pathway was significantly elevated with the increasing level of TNFRSF11B among tumors Normalized Enrichment Score (NES) = 1.814, *p* = 0.004) and in the comparison of tumors with paired colon epithelium (NES = 1.523, *p* = 0.027) (**Figures 5A, C, D**). TNFRSF11B also presented stronger transcriptional activity in tumor tissues than in paired colon epithelium (*p <*0.001, **Figure 5B**). Previous studies have revealed that pathogenic *E. coli* infection induces the overexpression of CEACAM6 in colon cancer, which, in turn, facilitates pathogenic *E. coli* adhesion (24). We demonstrated that CEACAM6 expression was significantly upregulated in cancer tissues that had high TNFRSF11B expression (**Figure 5E**). In addition, only six genes (KRT18, ARPC5L, ACTG1, ARPC2, EZR, and YWHAZ) related to pathogenic *E. coli* infection were simultaneously increased in both GSEA studies; these genes are mostly involved in focal adhesion, as determined *via* GO analysis (**Figures 5F, G**). Several KEGG pathways, such as regulation of actin cytoskeleton, shigellosis, bacterial invasion of epithelial cells, and Salmonella infection, were also affected by these six genes (**Figure 5H**). Next, we attempted to determine the interaction between TNFRSF11B expression and these six genes *via* hub gene analysis. TNFRSF11B may affect the transcriptional activity of YWHAZ to regulate pathogenic *E. coli* infection (**Figure 5I**), which was further validated in the TCGA-COAD database, in which increased TNFRSF11B expression correlated with increased TWHAZ expression (cor = 0.185, p <0.001) (**Figure 5J**).

### TNFRSF11B Decreased Central and Effector Memory CD4+ T Cell Infiltration Into the Colon Cancer Microenvironment

**Figure 6:**
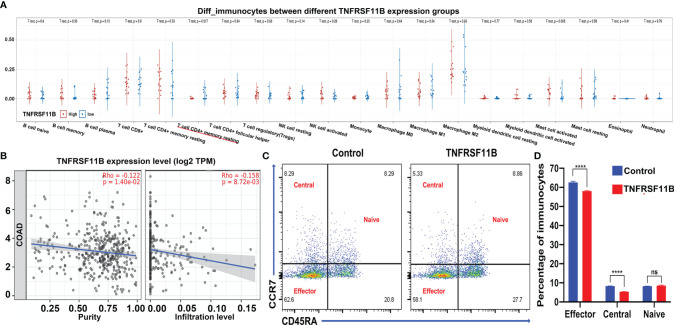
Immunocyte analysis and validation by PBMC stimulation. The subgroup of immunocytes in our RNA-sequencing samples was further analyzed *via* CIBERSORT algorithms **(A)**; as a result, only memory CD4^+^ T cells were activated in the colon microenvironment. The correlation between memory CD4^+^ T cell activation and the expression of TNFRSF11B in the TCGA-COAD project is also shown **(B)**. Accompanying the addition of soluble TNFRSF11B to PBMC culture medium, fluorescence-activated cell sorting (FACS) revealed different proportions of subgroups of memory CD4^+^ T cells **(C)**, and the bar chart shows the results **(D)**. ns, not significant; ****p < 0.0001.

To analyze the potential role of TNFRSF11B in the regulation of the immune response, we determined the different subsets of immunocytes between different TNFRSF11B-expressing colon cancer tissues in our cohort *via* CIBERSORT algorithms. Only activated memory CD4^+^ T cells (*p* = 0.017) were significantly decreased in the high TNFRSF11B expression group (**Figure 6A**), which was supported by the findings in the TCGA-COAD dataset. Using the TIMER 2.0 database, we also demonstrated that TNFRSF11B negatively affected the infiltration of activated memory CD4^+^ T cells into the colon cancer microenvironment (cor = −0.158, *p* = 0.008) (**Figure 6B**). We also confirmed the suppressive role of TNFRSF11B in the regulation of central memory CD4^+^ T cells and effector memory CD4^+^ T cells *via* the addition of soluble TNFRSF11B to PBMC culture medium. TNFRSF11B dramatically downregulated the immune activity of central memory CD4^+^ T cells and effector memory CD4^+^ T cells (all *p <*0.001) (**Figures 6C, D**).

## Discussion

Recently developed immunotherapeutic strategies for colon cancer, including ICIs and CAR-T cells, have been unsuccessful for many patients due to the low level of immune cell infiltration into the tumor microenvironment and effector T cell exhaustion caused by Treg cell and MDSC suppression (25). Memory CD4^+^ T cells are well characterized as having lower activation thresholds, an enhanced capacity to migrate to lymph nodes, long survival times, and reduced susceptibility to suppression by Treg cells and are the primary immune surveillance cells of the colon mucosa; thus, memory CD4+ T cells, rather than effector T cells, have become the focus of immunotherapy for colon cancer (26). Methods for activating memory CD4^+^ T cells and mediating their antitumor ability are urgently required. In our study, we observed the immunosuppressive role of TNFRSF11B in the regulation of memory CD4+ T cell activation in colon cancer tissue and peripheral blood monocytes, and our findings were validated in TCGA-COAD datasets. A negative correlation was observed between TNFRSF11B expression levels and the infiltration of activated memory CD4^+^ T cells into the tumor microenvironment using this dataset.

Pathogenic bacterial invasion leads to an immune response in the colon mucosa *via* M cells that mediate pathogen presentation and DCs, which present pathogenic antigens to activate the differentiation of effector T cells and eliminate bacteria (27). Consequently, bacteria-specific memory T cells differentiate from effector T cells while clearing pathogenic bacteria. Dysbiosis of the gut microbiota hampers the anticancer effect of CTLA-4 Ab in the MC38 colon cancer model. Moreover, treatment using Bacteroidales (Bf), an immune-stimulating adoptive transfer of memory Bf-specific TH1 cells or transplantation feces from a CTLA-4-responsive donor, partly restored CTLA-4 Ab efficacy (28). The amplification and diversity of the colonic microbiota are closely related to colon cancer progression and response to immunotherapy. Recently, pathogenic pks-positive *E. coli* strains have emerged as risk factors for colon cancer, as revealed by previous studies (29–31). These strains of *E. coli* produce colibactin, which alkylates DNA and contributes to colon cancer development (32). Developing a method to block intestinal pathogenic bacteria-related host gene alterations and recover the microbiota of the colon mucosa to restore immunity is an important future focus for the cancer immunotherapy field.

TNFRSF11B correlates with colonic mucosal immunity and is increased in the presence of *E. coli* infection resulting in diarrhea, and the potentiation of Peyer’s patch M cell self-renewal ability to ameliorate the inflammatory response can facilitate *Salmonella* infection (7, 33). Our findings showed that only pathogenic *E. coli* infection was altered in tumor tissues and tumors highly expressing TNFRSF11B *via* a specific GSEA approach. CEACAM6 expression was dramatically upregulated in TNFRSF11B-expressing tumors, which may facilitate pathogenic *E. coli* adhesion. In addition, six genes, KRT18, ARPC5L, ACTG1, ARPC2, EZR, and YWHAZ, were simultaneously enriched in tumors and tumor tissues highly expressing TNFRSF11B, which may enhance pathogenic *E. coli* focal adhesion, actin filament binding, and cadherin binding, as demonstrated by GO functional analysis. Similarly, these six genes were also involved in KEGG pathways related to bacterial adhesion and infection, including regulation of actin cytoskeleton, shigellosis, bacterial invasion of epithelial cells and *Salmonella* infection. However, elucidation of the sequelae of colorectal cancer progression and *E. coli* infection still requires more *in vitro* and *in vivo* experiments. TNFRSF11B and YWHAZ are both involved in the regulation of gastric cancer prognosis (34), and we found that the expression of YWHAZ, a transcription factor, was upregulated in tumors highly expressing TNFRSF11B, which was also supported by the finding of a strong correlation between TNFRSF11B and YWHAZ expression in the TCGA-COAD dataset. We hypothesized that TNFRSF11B may regulate YWHAZ transcriptional activity to affect tumorigenesis in colon cancer.

TNFSF11 could trigger the proliferation of lymph node stroma and activation of mesenchymal lymphoid tissue organizer cells when cooperating with TNFSF11A, which is specifically inhibited by TNFRSF11B (6). TNFRSF11B binding with TNFSF11 suppresses the recruitment of macrophages to lymph nodes and the migration of T cells (35), which indicates that TNFRSF11B enhances tumor lymph node invasion. Meanwhile, TNFRSF11B activated the phosphorylation of GSK-3β and enhanced the expression of β-catenin and its downstream effectors to strengthen the invasiveness of gastric cancer (26). In this study, after accurate systematic analysis of TCGA-COAD immune genes and development of the formula to predict survival outcomes, we ultimately found that TNFRSF11B significantly affected tumorigenesis, especially LN status and TNM stage, and influenced long-term survival rates in colon cancer. Using a single-cell database, we also demonstrated that TNFRSF11B affected the terminal phase of cell differentiation, metastasis, angiogenesis and the inflammatory response in colon cancer. In our IHC sample dataset, we also showed that TNFRSF11B was closely correlated with LN metastasis, LVI status and survival outcome. All these findings indicated that TNFRSF11B may promote lymph node metastasis in colon cancer.

This is a highly repeatable bioinformatics study, and four independent datasets were involved, including public databases and our single-cohort center. At the protein and transcriptional levels, we attempted to illuminate the role of TNFRSF11B in the regulation of the colon cancer immune microenvironment. We hypothesized that the expression of TNFRSF11B in colon cancer may be regulated by pathogenic *E. coli*. Infection may affect memory-activated CD4+ T cell infiltration and lymph node invasion. In this study, we explored the mechanism by which TNFRSF11B remodels the colon cancer immune response *via* bioinformatics analysis and confirmed the results with *in vitro* experiments; however, more biological experiments are needed to validate our findings.

## Data Availability Statement

The datasets presented in this study can be found in online repositories. The names of the repository/repositories and accession number(s) can be found below: NCBI SRA database, SUB10070043.

## Ethics Statement

The studies involving human participants were reviewed and approved by the Institutional Review Board of the Fujian Medical University Union Hospital. The patients/participants provided their written informed consent to participate in this study. Written informed consent was obtained from the individual(s) for the publication of any potentially identifiable images or data included in this article.

## Author Contributions

Conceptualization: J-RZ, PH, and X-QC. Data curation: J-RZ, X-JW, Z-YH, and HW. Formal analysis: J-RZ, PH, Z-QW, and X-CS-G. Funding acquisition: J-RZ, PH, and X-QC. Investigation: J-RZ, PH, FY, and B-QL. Methodology: J-RZ, PH, X-QC, X-JW, and Z-YH. Project administration: X-QC, B-QL, and Z-YH. Resources: J-RZ, PH, X-QC, and X-JW. Software: J-RZ and X-JW. Supervision: X-QC, B-QL, and Z-YH. Validation: PH, Z-QW, and X-CS-G. Visualization: J-RZ and X-JW. Roles/writing—original draft: J-RZ, PH, and X-QC. Writing—review and editing: J-RZ, Z-YH, and X-QC. All authors contributed to the article and approved the submitted version.

## Funding

This work was supported by the Joint Funds for the Innovation of Science and Technology, Fujian province [grant numbers: 2018Y9006 to Jun-rong Zhang, 2018Y9054 to Xian-qiang Chen]; Startup Fund for Scientific Research, Fujian Medical University [grant number: 2019QH1016 to Ping Hou]; Education Department of Fujian Province Young and Middle-aged Teacher Education Research Project [grant numbers: JAT190180 to Ping Hou, JT180181 to Jun-rong Zhang].

## Conflict of Interest

The authors declare that the research was conducted in the absence of any commercial or financial relationships that could be construed as a potential conflict of interest.

## Publisher’s Note

All claims expressed in this article are solely those of the authors and do not necessarily represent those of their affiliated organizations, or those of the publisher, the editors and the reviewers. Any product that may be evaluated in this article, or claim that may be made by its manufacturer, is not guaranteed or endorsed by the publisher.

## Supplementary Material

Supplementary Figure 1 Expression of TNFRSF11B in normal lymph nodes. The transcriptional levels of TNFRSF11B in different tissues from the Harmonizome database were shown in the histogram **(A)**. Validation of the expression of TNFRSF11B at the protein level in in germinal center cells and non-germinal center cells **(B–D)** by the Human Protein Atlas database (IHC staining) **** indicates a P value<0.0001.

Supplementary Figure 2 Study workflow.
