# Supplementary figures and images for: TNFRSF11B Suppresses Memory CD4+ T Cell Infiltration in the Colon Cancer Microenvironment: A Multiomics Integrative Analysis
Source: Front Immunol. 2021 Dec 6;12:742358. doi: 10.3389/fimmu.2021.742358 (PMC8685235; doi:10.3389/fimmu.2021.742358)

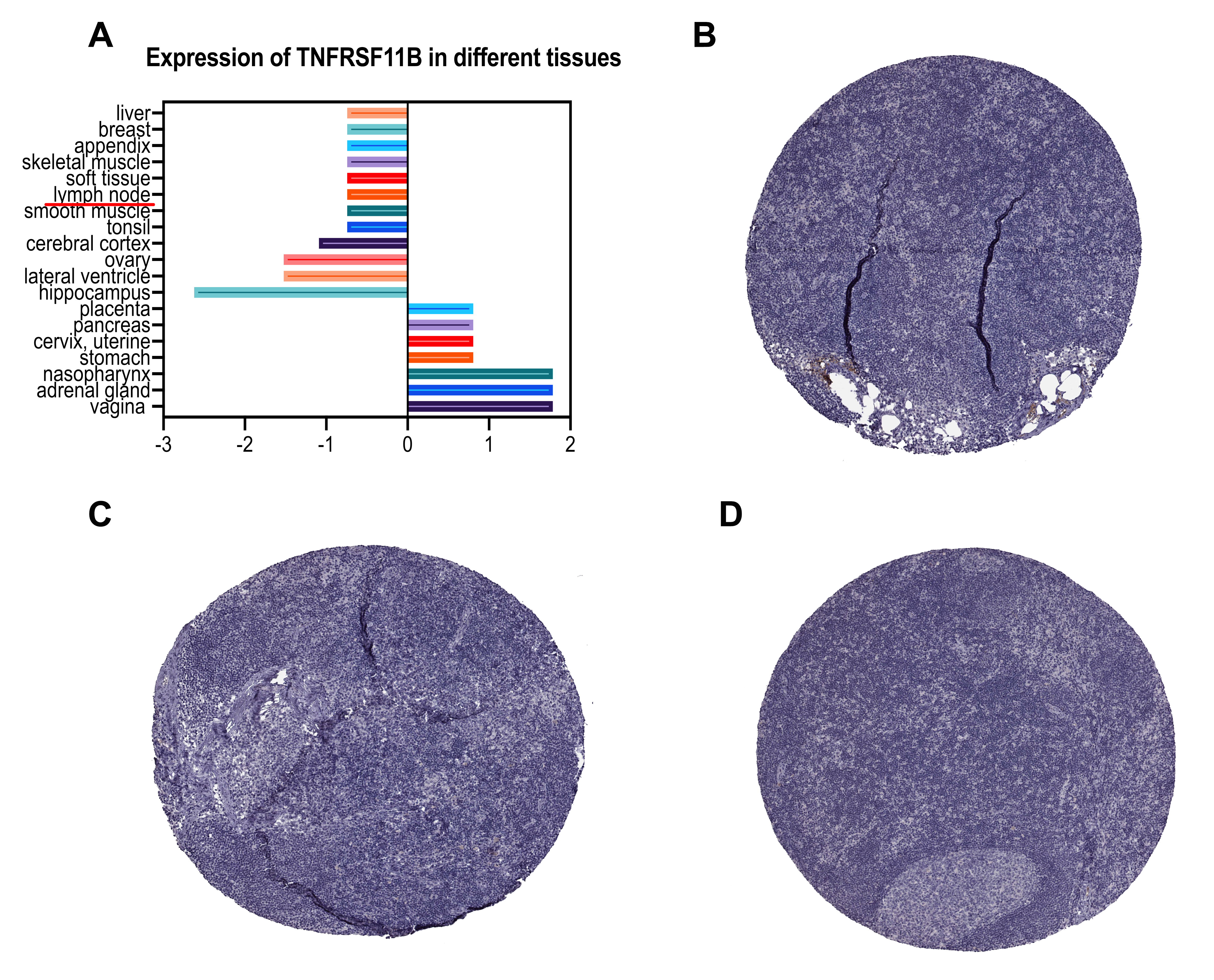

Supplement: Supplementary Figure 1 — Expression of TNFRSF11B in normal lymph nodes. The transcriptional levels of TNFRSF11B in different tissues from the Harmonizome database were shown in the histogram (A). Validation of the expression of TNFRSF11B at the protein level in in germinal center cells and non-germinal center cells (B–D) by the Human Protein Atlas database (IHC staining) **** indicates a P value<0.0001. [file Image_1.tif]

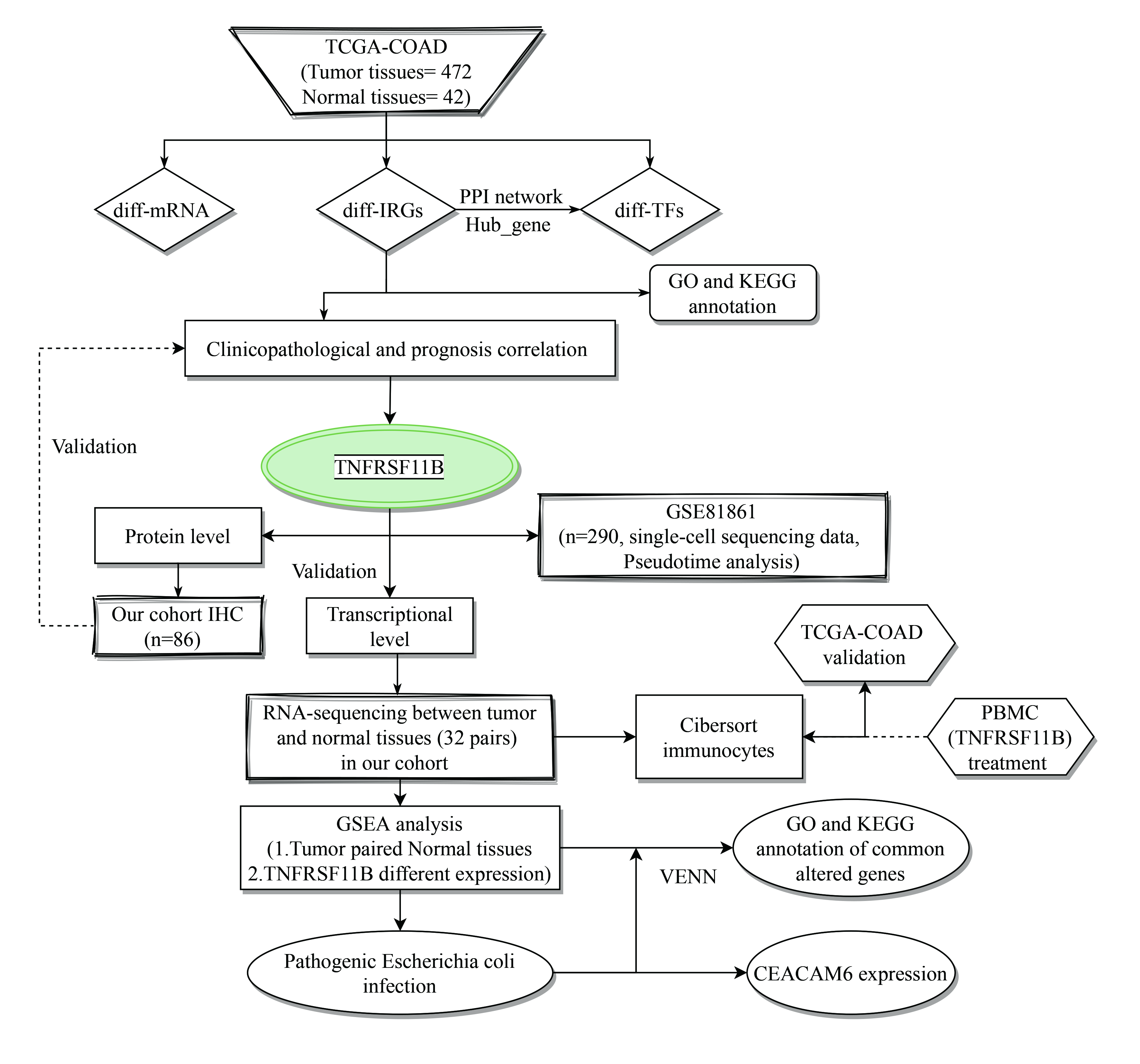

Supplement: Supplementary Figure 2 — Study workflow. [file Image_2.tif]
